# Supplementary material for: Plant response to intermittent heat stress involves modulation of mRNA translation efficiency
Source: Plant Physiol. 2024 Dec 17;197(2):kiae648. doi: 10.1093/plphys/kiae648 (PMC11979764; doi:10.1093/plphys/kiae648)
Supplement: kiae648_Supplementary_Data [file kiae648_supplementary_data.zip › suppfigs.pdf]

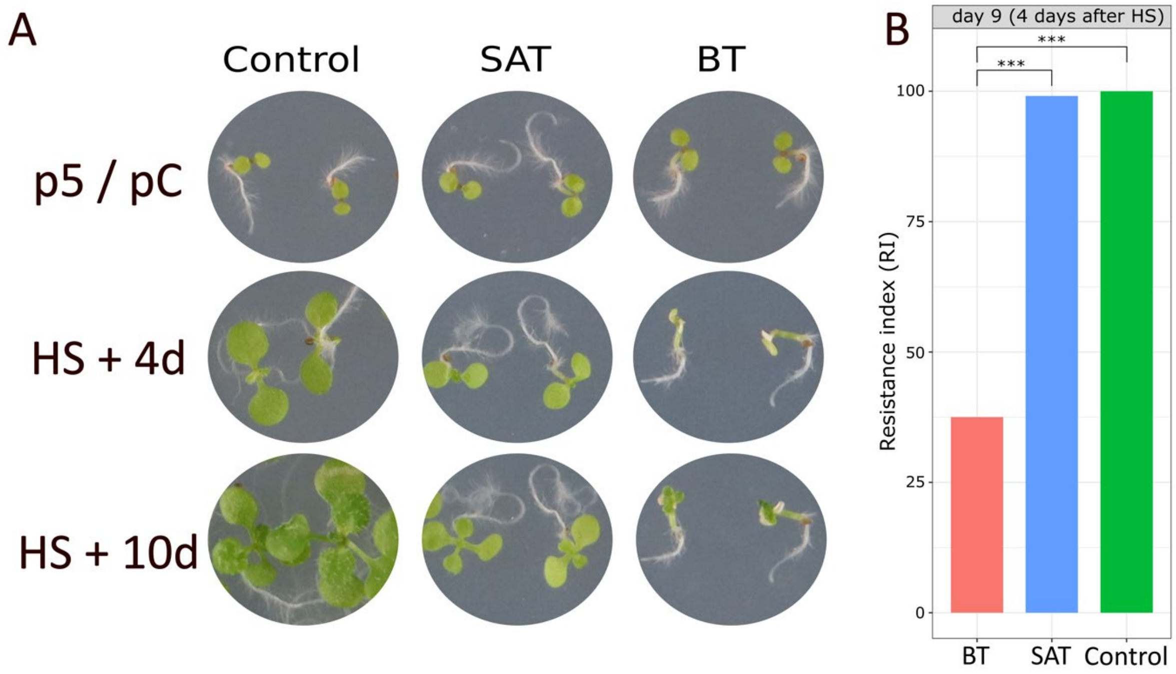

Supplementary Figure S1. **Effect of priming on Arabidopsis seedling phenotypes.** A) For the short acquired thermotolerance (SAT) and basal thermotolerance (BT) heat stress regimes, images were taken 7.5 h (p5/pC of Figure 1), 4 days and 10 days after exposure to 44°C. The control condition (left) is plants maintained at 20°C for the same time periods. B) Measurement of the Resistance Index 4 days after BT (red), SAT (blue) and control (green) conditions. The Resistance Index (RI) is measured as the percentage of plants showing no bleaching effect 4 days after exposure to 44°C, such that a high RI indicates greater resistance and a low RI indicates that plants are more susceptible to the stress. Nine biological replicates were used for each condition, with each plate containing 50 seeds, for a total of 450 seeds per condition. Wilcoxon tests were used to compare all three conditions (*P< 0.05, **P< 0.01, ***P< 0.001). Only significant variations are presented.

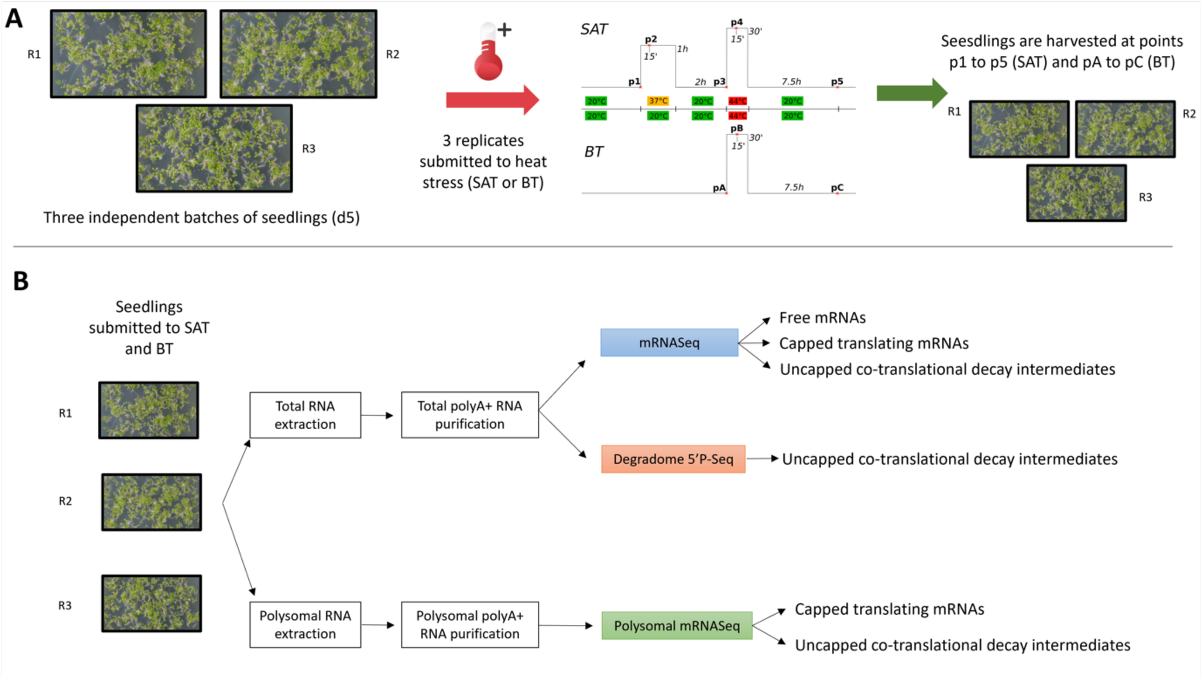

Supplementary Figure S2. Visual summary of the experimental procedure. A) Three batches of seedlings were grown *in vitro*, which served as three independent biological replicates (R1 to R3). On day 5 (d5), two heat stress treatments were applied: short acquired thermotolerance (SAT, priming) or basal thermotolerance (BT, no priming). Seedlings were harvested at different time points during the heat stress treatments (p1 to p5 and pA to pC). B) For each sample collected, two types of RNA extractions were performed to obtain total or polysomal RNAs (the latter isolated by sucrose gradient fractionation). PolyA+ mRNAs were then purified from the total RNA fractions and a classical mRNA-seq or a degradome 5'P-seq protocol was applied. The mRNA-seq approach allows the sequencing of free mRNAs, capped translating mRNAs and uncapped co-translational decay intermediates, whereas the degradome 5'P-seq strategy is specific for uncapped co-translational decay intermediates. PolyA+ mRNAs were also purified from the polysomal RNA fractions and sequenced to generate the polysomal mRNA-seq library containing both capped translating mRNAs and uncapped co-translational decay intermediates.

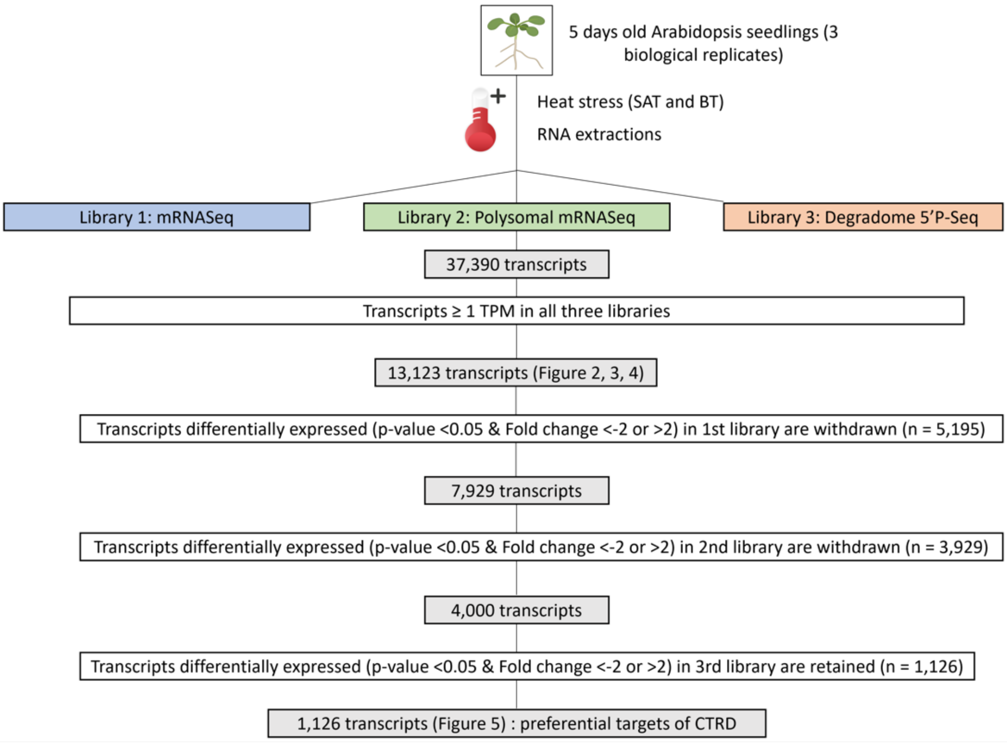

Supplementary Figure S3. Experimental design and filtering strategy used to generate Figures 2 to 5. Three libraries were generated using different NGS techniques (see Supplementary Figure 2). A first filter was used to retain only transcripts with one or more TPM in all three libraries, generating a new dataset of 13,123 transcripts. The degradome 5'P-seq data was used to generate Figure 2 and the three datasets were used to generate Figure 3 and 4. To generate Figure 5, all transcripts differentially expressed (p-value <0.05 & FC >2 or <-2) in libraries 1 and 2 were removed, resulting in a dataset of 4,000 genes. Then, only transcripts differentially expressed in library 3 were retained and considered as preferred targets of cotranslational decay (CTRD).

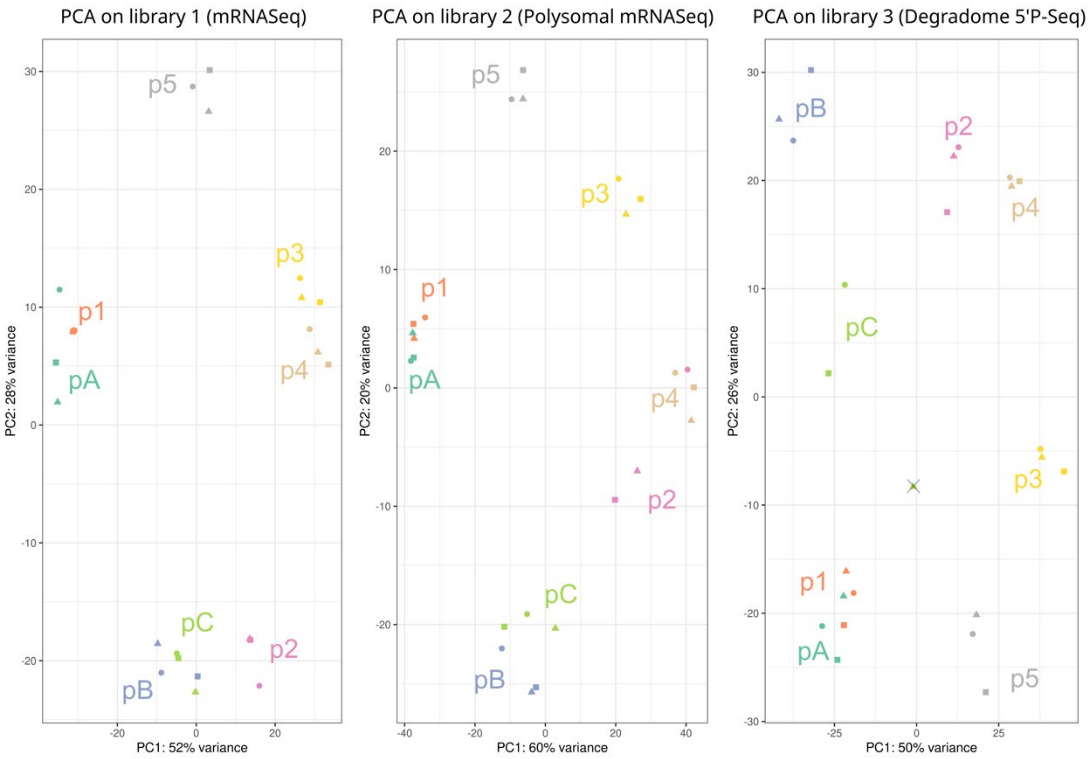

Supplementary Figure S4. Library quality control. Three two-dimensions principal component analysis (PCA) were conducted for each library produced (A : library 1, B : library 2 and C : library 3). The two axes can explain 80%, 80 and 76% of the variance for each library respectively. Each replicate is represented by round (R1), triangle (R2), square (R3). In "C", the second replicate of pC diverged from the other two and was withdrawn from the study (represented by a cross on the figure).

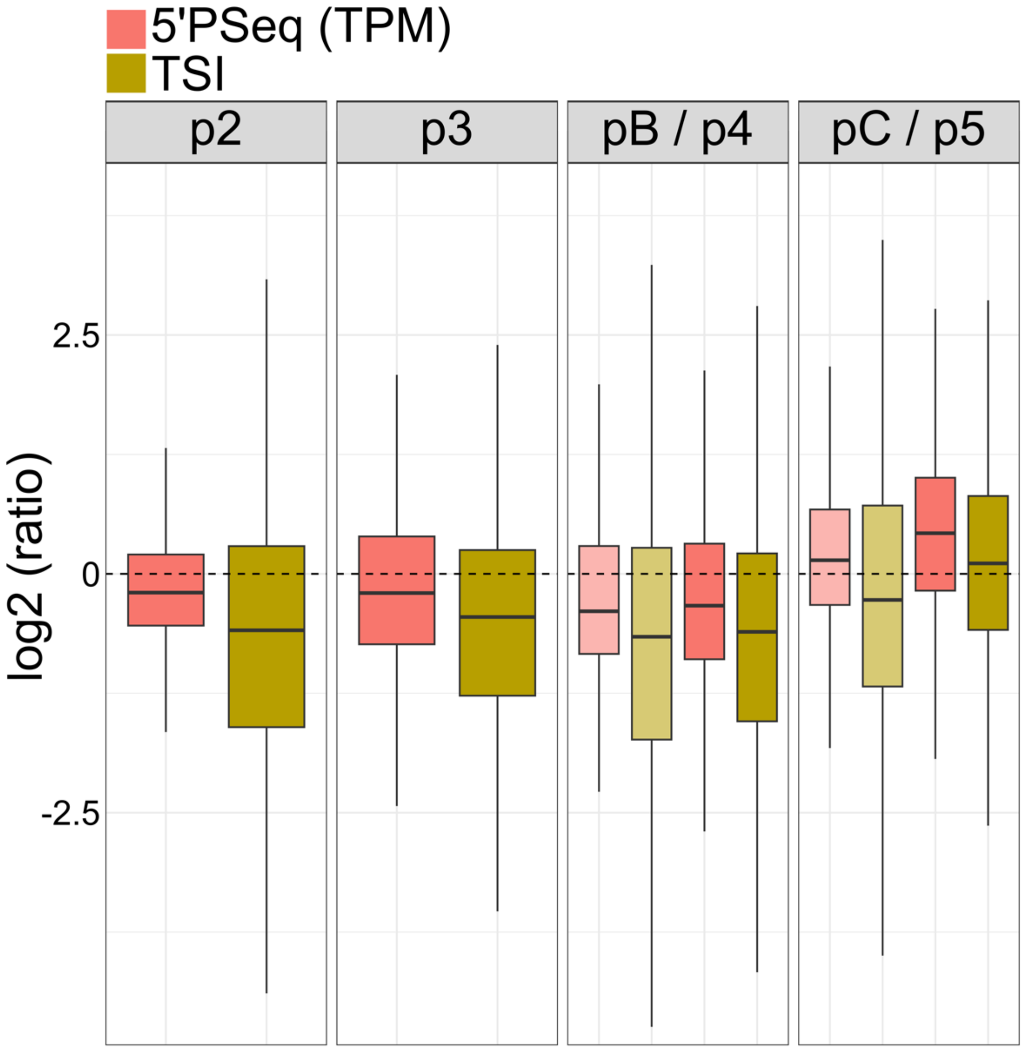

Supplementary Figure S5. Comparison of the distribution of the ratio at different short acquired thermotolerance (SAT) and basal thermotolerance (BT) time points (compared to the p1 situation) of total degradome reads (shown in light and dark red boxplots) to the ratio of reads found exactly at position 16-17nt before the stop codon (as measured by the translational termination stalling index (TSI), shown in light and dark green boxplots) for the genes of Figure 3. With the exception of the pC time point of the BT heat stress regime, which has a smaller number of 16-17 nt reads (as shown in Figure 2C), the compared distributions follow the same trends, supporting the conclusion that cotranslational decay (CTRD) products dominate the global degradome reads. For each boxplot, the center line represents the median, the lower and upper limit of the boxes represent respectively the first and third quartiles. The whiskers represent 1.5 fold of the interquartile range. Outlier points are not represented. For the total degradome N=13,123 at all points while for the TSI N=5222 for p1, 4217 for p2, 5442 for p3, 4625 for p4, 5856 for p5, 4222 for pB and 4149 for pC.

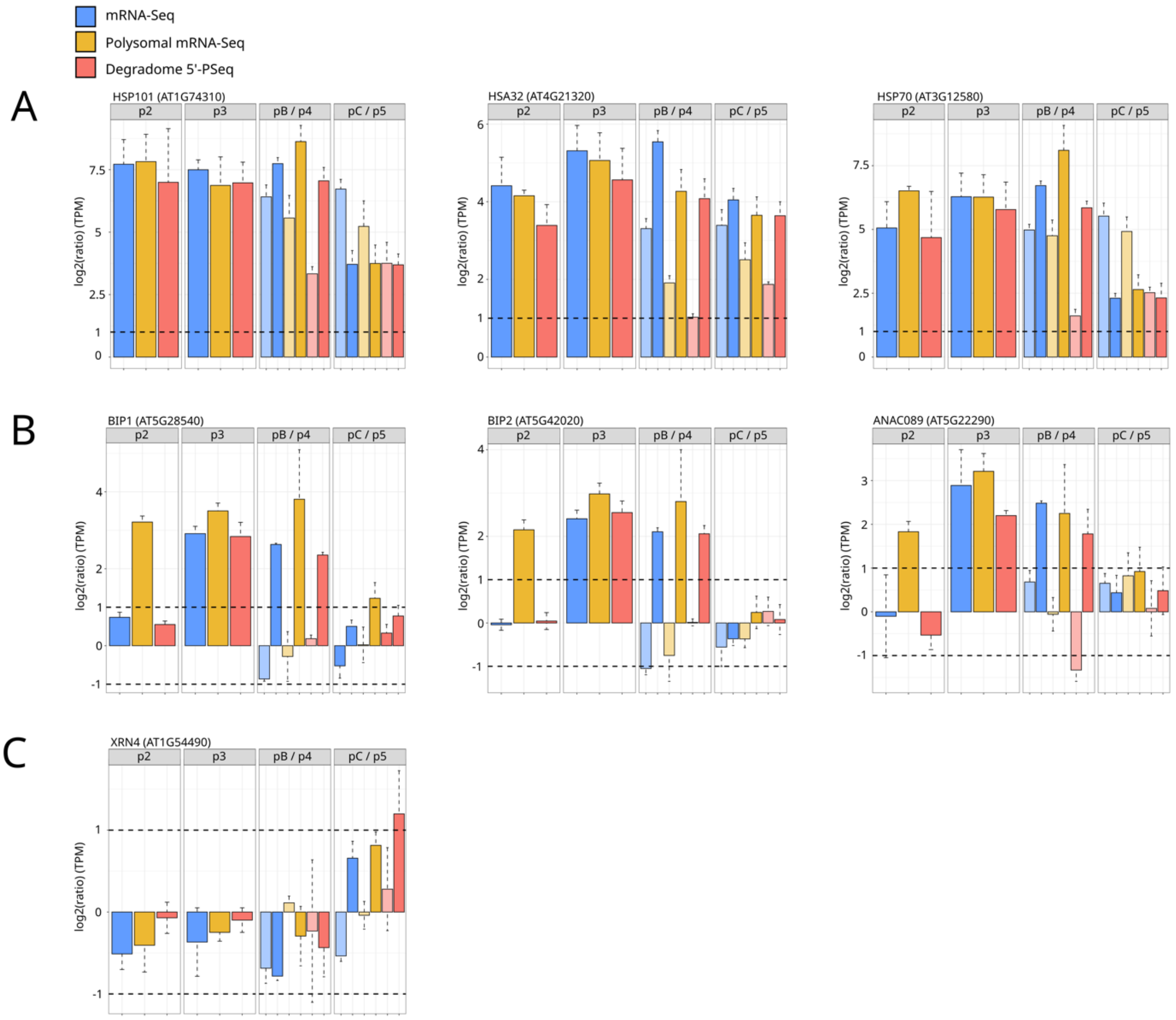

Supplementary Figure S6. Priming is required to induce key members of the Unfolded Protein Response (UPR) pathway, while both basal thermotolerance (BT) and short acquired thermotolerance (SAT) induce the cytosolic protein response (CPR) pathway. A) Histograms showing the distribution (log2(TPM ratio)) of key members of the CPR pathway: HSP101, HSA32, HSP70 in the three databases at different SAT and BT time points compared to 20°C situation. B) Histograms showing the distribution (log2(TPM ratio)) of key members of the UPR pathway: BIP1, BIP2, ANAC089 at different SAT and BT time points compared to 20°C situation. C) Histograms showing the distribution (log2(TPM ratio)) of XRN4 at different SAT and BT time points compared to 20°C situation. Error bars represent the standard deviation (SD) of three biological replicates. For A, B and C the distribution of total mRNAs is shown in blue, polysome-associated mRNAs in brown and cotranslational decay (CTRD) products in red. Pale colored boxplots represent unprimed conditions (pB and pC) and dark colored boxplots represent primed conditions (p4 and p5)
