# Supplementary material for: Plant response to intermittent heat stress involves modulation of mRNA translation efficiency
Source: Plant Physiol. 2024 Dec 17;197(2):kiae648. doi: 10.1093/plphys/kiae648 (PMC11979764; doi:10.1093/plphys/kiae648)
Supplement: kiae648_Supplementary_Data [file pp.02392.2024-s07.pdf › pp.02392.2024-s07.pdf]

Plant Physiology®

Plant response to intermittent heat stress involves modulation of mRNA translation efficiency

Arnaud Dannfald, Marie-Christine Carpentier, Rémy Merret, Jean-Jacques Favory, and Jean-Marc Deragon

Review Timeline:

Submission Date:	24-Jan-2024
Editorial Decision:	25-Mar-2024
Resubmission Received:	04-Sep-2024
Editorial Decision:	25-Oct-2024
Revision Received:	06-Nov-2024
Accepted:	11-Nov-2024

25-Mar-2024

Prof. Jean-Marc Deragon
1- CNRS LGDP-UMR5096, 58 Av. Paul Alduy 66860 PERPIGNAN, FRANCE. 2- Université de Perpignan Via Domitia, LGDP-UMR5096, 58 Av. Paul Alduy 66860 PERPIGNAN, FRANCE.
Perpignan 66860
France

RE: Plant response to intermittent heat stress involves modulation of mRNA translation efficiency

Dear Dr. Jean-Marc Deragon:

Thank you for giving us the opportunity to review your manuscript for publication in the Plant Physiology. The reviewers have found the manuscript of interest, but have raised a number of concerns that will require your attention. As these concerns are of some gravity and will entail substantial changes to the manuscript, you are likely to need additional time to address them. I must therefore reject the manuscript in its present form. Nonetheless, if you are able to address the points raised in review, I would welcome your resubmission of a manuscript that incorporates your responses.

I hope the reviewers' detailed comments will be informative if you decide to resubmit following a significant revision addressing all points that reviewers listed as lacking clarity on methods and results, absence of certain controls, and alternative possibilities for current observations. The key aspects to address include an improved comparative analysis with clarifying details and relevant justifications, and incorporation of the published work to convincingly state how this work adds novelty to the field.

Please note that any resubmission will be treated as a new entry to the system and is likely to go out for review.

If you do have any queries or concerns, you are welcome to contact me in the first instance.

Thank you again for allowing Plant Physiology to review your work. We look forward to hearing from you soon.

Sincerely,

Editor, Plant Physiology

-- Reviewer comments:
Reviewer #1 (Comments for the Author):

In the manuscript entitled "Plant response to intermittent heat stress involves modulation of mRNA translation efficiency" by Dannfald, et al., the authors investigated the relationship between translation and co-translational decay (CTRD) in response to heat stress. They monitored mRNA abundance, translatome, and degradome in response to heat stress with or without priming. The authors concluded that (i) priming preserves the global translational potential and differentially affects the translational efficiency of stress and non-stress-related genes; (ii) priming is essential for up-regulating the UPR pathway and protecting ER-associated mRNA from CTRD; and (iii) conclude that CTRD acts independently during heat stress to regulate the expression of a subset of genes.

Major Comments:
Overall I think the authors can provide more details for the rationale for some experiments and also more details on some of the comparisons performed. For example, for SAT, the recovery period was 7.5h post-stress while for BT it was 2h. It seems that to compare the effects of priming the recovery time points should be the same to assess how the gene expression dynamics recovers. This might also help to explain the observation in Figure 2C, "After 7.5h at 20{degree sign}C without priming, translation is still mainly down (pC), but is functional again at p5, although at a lower level than the initial 20{degree sign}C situation (Figure 2C)."

It has been shown that most of the translation recovers within 1h. There seems to still be a significant decrease in the amount of the 16-17nt peak upstream of the stop sites which is surprising. Can the authors provide more insights on this observation?

There are several published transcriptome and translatome work that the authors can leverage on heat stress for some comparisons and insights, particularly to examine key marker genes for heat stress and translation (Zhang L, et al., https://doi.org/10.1105/tpc.16.00808; Bonnot T et al., 10.1093/plcell/koab113).

The images in Figure 1B make it difficult to confidently conclude the phenotypes. The authors should perform a more

quantitative measure.

In Figure 4C, it would be great if the authors provided the individual profiles for a few specific genes. Such as UPR-related genes mentions in Figure 5 and HSPs and Translation factors. Marker genes for each expected response in these datasets. This will also support the author's conclusion on Lines 562- 563 "This cannot occur in BT because the UPR pathway is not stimulated in this condition, leading to a higher level of CTRD".

In Figure 5, Lines 563 - 567, the authors stated that "We also observed that the situation described for cluster 4 mRNAs, which are likely to be regulated only by CTRD in SAT/BT (N=259), can be extended to all mRNAs translated in the RE (N= 944) (Figure 5C). In support of this model, we observed that XRN4, the major enzyme responsible for CTRD (Merret et al., 2015), is present at higher levels in the BT polysome at 44{degree sign}C compared to SAT (Figure 5F and G). What about XRN4 levels in the transcriptome, polysome, and degradome dataset in SAT vs BT?

To support the observation shown in the western blot, the authors need to include a loading control.

In the Methods, Lines 702 - 706 explaining the Heat map, hydrophobicity, and HSP70 scores, could the authors provide details on where the targets of XRN4 were obtained?

Minor Comments
Overall, the fonts for most of the figures should be made larger. For some maximizing the zoom results in blurred text.
Fig 1, Panel A, it is not clear what is S+U.
Figure 1 Legend. Explain 4j and 8j as shown in 1B.
Line 455, 458, and 566 should be ER and not RE
Several grammatical errors throughout the text should be corrected.

Reviewer #2 (Comments for the Author):

Dear Editor,
The manuscript entitled "Plant response to intermittent heat stress involves modulation of mRNA translation efficiency" prepared by Dannfald et al demonstrated that a short term and mild heat stress (heat priming) would affect the transcriptome, translatome and degradome of Arabidopsis seedlings, and finally resulting in translation efficiency of the seedlings when suffering another round of heat stress. These polysome-related processes of mRNA enabled the primed plant to cope better to severe heat stress than those without priming. By comprehensive analyses of sequencing data, they also found that co-translational mRNA decay (CTRD) can specifically regulate a great number of genes during heat stress, and suggested that CTRD could be considered as an independent gene regulatory mechanism in plants. The study is interesting and has its novelty, provides us the regulations of mRNA level and usage in polysome-related processes when plants under different high temperature and recovery conditions. Thus, I have two comments here:
1. Could they please explain the standard in setting the time point for sample collection? Do they have any reference or base on personal experience? Why p2 was 15min minutes after heat priming? Also, p4 was set at 15 minutes after heat stress. In my opinion, collect the samples at the end of each treatment could present the effect of the treatment on mRNA metabolism more accurately.
2. Have they ever considered why mRNA transcripts of different genes regulated differently during these polysome-related processes? I think an addition of related analysis or, at least in depth discussion in this point should improve the quality of the manuscript a lot.

Reviewer #3 (Comments for the Author):

In this study, Dannfald et al. conducted RNA-seq, polysome profiling, and 5'P seq analyses to elucidate plant responses to heat stress at the translational level with or without a priming event. The authors discovered that the priming event aids in restoring mRNA translation post-heat stress, and heat stress-responsive mRNAs exhibit increased translation efficiency compared to non-heat-responsive genes under heat-stressed conditions. Furthermore, the priming event triggers a set of genes related to the UPR pathway, while ER-translated mRNAs undergo cotranslational mRNA decay in the absence of priming. However, the manuscript is overly descriptive and lacks clarity, making the findings less appealing for publication in Plant Physiology.

Several concerns regarding the manuscript are as follows:
1) The calculation of translation efficiency is a major concern. The authors define TE as the ratio of polysomal mRNA-seq to degradome (5'P-seq) data, which may not accurately reflect TE. It is suggested that TE should be calculated as the difference between polysomal mRNA-seq and degradome over mRNA-seq, i.e., (polysomal mRNA-seq - degradome) / mRNA-seq. This discrepancy raises questions about the accuracy of TE calculation.
2) The relationship between RNA-seq, polysome profiling, and 5'P seq meta-analyses is not clearly explained in the manuscript, leading to confusion about how these analyses are interconnected.
3) Experimental validation of proposed TE or CTRD changes is missing from the manuscript, making the findings less robust.

4) According to the Figure5F and G, the abundance of XRN4 is increased under BT conditions, correspondingly, CTRD would be stronger. However, in Figure 3A, the situation contradicts with the hypothesis, need clarification to address inconsistencies in the data.
5) Exploring mutants of HSP70 and XRN4 to analyze changes in TE efficiency and CTRD could enhance the manuscript's quality.

Minor concerns:
1) In the SAT conditions, the priming treatment lasts for 1 hour. Why did the authors collect tissues at 15 minutes after heat stress? Would it be more appropriate to collect tissues at the end of the priming event? A similar question arises for the P4 and PB conditions.
2) The figure legends should include more detailed information, particularly for the abbreviations used. For example, "GMUCT" in Figure 4 and the labeling in Figure 1B (4j? 8j?) should be clarified (e.g., 4d? 8d?). Additionally, statistical analysis should be provided for Figure 1B.
3) There are numerous errors in the figures, such as Figure 3A where the values on the vertical axis are off-center. The descriptions like "* = p-value < 0.05, ** = p-value < 0.01, *** = p-value < 0.001" are not appropriate and should be revised.
4) In Figure 4A, it displays 729 genes, while in line 310, it states "N=730." This inconsistency should be addressed and corrected.
5) There are discrepancies in the descriptions regarding data selection. For instance, in one section it mentions retaining mRNAs with at least 1 TPM in all conditions, while in another section, it refers to a filter applied to the original dataset of 37390 genes. These inconsistencies need to be clarified and resolved for coherence.

Reviewer #1 (Comments for the Author):

In the manuscript entitled "Plant response to intermittent heat stress involves modulation of mRNA translation efficiency" by Dannfald, et al., the authors investigated the relationship between translation and co-translational decay (CTRD) in response to heat stress. They monitored mRNA abundance, translatome, and degradome in response to heat stress with or without priming. The authors concluded that (i) priming preserves the global translational potential and differentially affects the translational efficiency of stress and non-stress-related genes; (ii) priming is essential for up-regulating the UPR pathway and protecting ER-associated mRNA from CTRD; and (iii) conclude that CTRD acts independently during heat stress to regulate the expression of a subset of genes.

Major Comments:
Overall I think the authors can provide more details for the rationale for some experiments and also more details on some of the comparisons performed. For example, for SAT, the recovery period was 7.5h post-stress while for BT it was 2h. It seems that to compare the effects of priming the recovery time points should be the same to assess how the gene expression dynamics recovers. This might also help to explain the observation in Figure 2C, "After 7.5h at 20°C without priming, translation is still mainly down (pC), but is functional again at p5, although at a lower level than the initial 20°C situation (Figure 2C)."

We fully agree that to evaluate the effect of priming, the recovery time point should be the same in SAT (with priming) and BT (without priming). This is exactly what we did, as both recovery times (pC for BT and p5 for SAT) were performed 7.5 h after the 44°C heat stress (see Figure 1A). Therefore, it is not correct to say that the recovery period was measured 7.5h after the stress for SAT and 2h for BT, as both measurements were made 7.5h after the stress (pC and p5). Thus, we can effectively compare the state of translation 7.5h post-stress for SAT compared to BT. Using two different approaches (the amount of polysomes normalized to the total amount of ribosomes (Figure 1C) and the amount of the 16-17nt peak upstream of the stop codons in the 5'P-seq meta-analysis (Figure 2C)), we clearly show that priming is critical for translation recovery. Indeed, with priming, after 7.5h of recovery, translation is back to about 75% of its initial (20°C) level, while at the same time without priming, translation is still very low and identical to its 44°C level. We also provide in this new version more details on our workflow and filtering strategy in Supplemental Figure 2 and 3.

It has been shown that most of the translation recovers within 1h. There seems to still be a significant decrease in the amount of the 16-17nt peak upstream of the stop sites which is surprising. Can the authors provide more insights on this observation?

The time required for translation recovery is highly dependent on the intensity and duration of the heat stress applied. In this study, we apply a very stringent stress (44°C for 30 minutes) and we do so by immersing our plates in a water bath (as in Yeh et al., Plant Sci. 2012: 10-23) rather than simply placing the plates in a dry incubator at 44°C. The rationale for using this stringent condition is that it is more likely to reveal the putative importance of a previous priming step at 37°C. We agree with the reviewer that for a single 37°C heat stress for 1h (which may be the most commonly used heat stress condition), translation fully recovers after 1h. In fact, in Figure 1C (p3) we show full recovery after 2h following a 1h 37°C incubation. This is confirmed by the 16-17nt peak upstream of the stop codons (Figure 2A (p3)). However, after a much more severe heat stress (44°C for 30 minutes), priming is required for translation to recover even

after 7.5h of recovery time (Figure 1C and 2C). Thus, our experiment clearly demonstrated the importance of priming for translation recovery after severe heat stress.

There are several published transcriptome and translatome work that the authors can leverage on heat stress for some comparisons and insights, particularly to examine key marker genes for heat stress and translation (Zhang L, et al., https://doi.org/10.1105/tpc.16.00808; Bonnot T et al., 10.1093/plcell/koab113).

There are two main difficulties in performing the proposed comparisons. First, regarding the transcriptomic data, in this work we chose to work with only a subset of gene transcripts, those present in at least 1 TPM at all steps of the SAT and BT regimes and for all three databases (transcriptome, translatome and degradome). This strategy was chosen to be able to compare the behavior of these transcripts at all steps of a primed versus an unprimed regime, with the ultimate goal of evaluating the importance of priming on their translation efficiency. We did not aim to fully document all genes that would be transcriptionally up- or down-regulated following heat stress, as this has been done in many previous works. Therefore, a comparison of our transcriptomic data with those previously published would only lead to a partial overlap, since many genes (for example, those not present at 1 TPM at 20°C) were excluded from our data set.

The second difficulty concerns the previously published translatome data, which did not distinguish between polysomal mRNAs that are actively translated and polysomal mRNAs that are in the process of being degraded. They assumed that all polysome-associated mRNAs are necessarily actively translated. However, we have clearly shown in a previous study (Carpentier et al., Plant Physiology, 2020:1251-1262) that using a ratio of the normalized amount of mRNAs associated with polysomes to the normalized amount of mRNAs involved in the co-translational decay process is a better proxy for translation efficiency and corresponds very well to the final amount of protein produced. Also, we show here that cotranslational decay can be up or down without affecting the total amount of polysomal mRNAs detected. This means that simply measuring the total amount of mRNAs associated with polysomes (reported to total mRNAs) is not the best proxy for translation efficiency. This makes comparative analysis impossible because we are not measuring TE in the same way as before.

The images in Figure 1B make it difficult to confidently conclude the phenotypes. The authors should perform a more quantitative measure.

We agree with this comment and now provide quantitative measures of the impact of the BT and SAT heat stress regime on the plant recovery (Supplementary Figure 1B)

In Figure 4C, it would be great if the authors provided the individual profiles for a few specific genes. Such as UPR-related genes mention in Figure 5 and HSPs and Translation factors. Marker genes for each expected response in these datasets. This will also support the author's conclusion on Lines 562- 563 "This cannot occur in BT because the UPR pathway is not stimulated in this condition, leading to a higher level of CTRD".

As requested by this reviewer, we now present the profile of key genes involved in CPR and UPR responses in Supplementary Figure 5. This may help readers to visualize that priming is indeed required to induce the expression of UPR-related genes at 44°C (Supplementary Figure 5B), while this is not the case for CPR-related genes that are induced with or without priming (Supplementary Figure 5A).

In Figure 5, Lines 563 - 567, the authors stated that "We also observed that the situation described for cluster 4 mRNAs, which are likely to be regulated only by CTRD in SAT/BT (N=259), can be extended to all mRNAs translated in the RE (N= 944) (Figure 5C). In support of this model, we observed that XRN4, the major enzyme responsible for CTRD (Merret et al., 2015), is present at higher levels in the BT polysome at 44{degree sign}C compared to SAT (Figure 5F and G). What about XRN4 levels in the transcriptome, polysome, and degradome dataset in SAT vs BT?

We now present in (Supplementary Figure 5C) the variation of XRN4 transcript in all three databases (transcriptome, polysome and degradome). As can be observed in this figure, XRN4 transcript is not differentially accumulated in the three databases at any point of both heat stress regimes (SAT and BT), with a single exception (p5, up in the degradome database).

To support the observation shown in the western blot, the authors need to include a loading control.

The western blot of Figure 5F was performed using the same initial amount of biomass to evaluate the presence of XRN4 in the different input and polysome fractions. This means that much more proteins are present in the 20°C polysome fractions compared to the 44°C polysome fractions. In fact, we show in Figure 1 that there are much fewer polysomes at 44°C compared to 20°C, so for an equivalent biomass as starting material, we would expect to get much less protein in the 44°C polysome fractions compared to the 20°C polysome fractions. Despite this strong bias, XRN4 is not detected in the 20°C polysome fractions but is readily detected in the 44°C polysome fractions. We also observed fewer polysomes at 44°C in BT compared to SAT. Again, despite this bias leading to loading more protein with an equivalent biomass of SAT 44°C polysome fraction compared to BT, there is a clear increase of XRN4 in BT 44°C polysome fractions compared to SAT. Another way to do this analysis is to load the same amount of protein in each lane of the western blot instead of using the same amount of biomass. Using this approach, we can indeed provide a loading control by using a RPL13 antibody. This is what we did in the new Figure 5F. As can be seen, this confirm our initial results, showing that XRN4 are much more present in polysomes at 44°C in BT compared to SAT (Figure 5F and G).

In the Methods, Lines 702 - 706 explaining the Heat map, hydrophobicity, and HSP70 scores, could the authors provide details on where the targets of XRN4 were obtained?

We agree that this was not clearly stated in the Methods section. We used proteins from cluster 4 of Figure 5 to generate the hydrophobicity and HSP70 scores. Transcripts for these proteins are clearly preferential targets of CTRD at 44°C in BT (pB point, see heat map in Figure 5A). Since XRN4 is the major enzyme responsible for CTRD (Carpentier et al., Plant Physiology, 2020:1251-1262), these transcripts are likely targets of XRN4. To clarify the situation, we change the sentence "Protein sequences encoded by cluster 4 (which are targets of XRN4 in the pB state), to "Protein sequences encoded by cluster 4 (which are targets of CTRD at the pB step of BT)".

Minor Comments

Overall, the fonts for most of the figures should be made larger. For some maximizing the zoom results in blurred text.

We change the fonts of our figures to make sure that they are clearly visible.

Fig 1, Panel A, it is not clear what is S+U

Sorry for this. It was meant to indicate the position of free ribosomal subunits in the gradient. We change this for "SUB" and describe its signification in the figure legend.

Figure 1 Legend. Explain 4j and 8j as shown in 1B.

We apologies for leaving these French abbreviations for days (in French "jour"). We replaced "j" by "days" in Figure 1B

Line 455, 458, and 566 should be ER and not RE
We are sorry for these errors, which we have corrected.

Several grammatical errors throughout the text should be corrected.
We have carefully looked and corrected these grammatical errors (in red in the text)

Reviewer #2 (Comments for the Author):

Dear Editor,
The manuscript entitled "Plant response to intermittent heat stress involves modulation of mRNA translation efficiency" prepared by Dannfald et al demonstrated that a short term and mild heat stress (heat priming) would affect the transcriptome, translatome and degradome of Arabidopsis seedlings, and finally resulting in translation efficiency of the seedlings when suffering another round of heat stress. These polysome-related processes of mRNA enabled the primed plant to cope better to severe heat stress than those without priming. By comprehensive analyses of sequencing data, they also found that co-translational mRNA decay (CTRD) can specifically regulate a great number of genes during heat stress, and suggested that CTRD could be considered as an independent gene regulatory mechanism in plants. The study is interesting and has its novelty, provides us the regulations of mRNA level and usage in polysome-related processes when plants under different high temperature and recovery conditions.
Thus, I have two comments here:
1. Could they please explain the standard in setting the time point for sample collection? Do they have any reference or base on personal experience? Why p2 was 15min minutes after heat priming? Also, p4 was set at 15 minutes after heat stress. In my opinion, collect the samples at the end of each treatment could present the effect of the treatment on mRNA metabolism more accurately.

Indeed, we have a lot of experience in studying mRNA processes after heat stress (Merret et al, Cell Reports 2013, Merret et al, NAR, 2015, Merret et al, Plant Phys, 2017, Carpentier et al, Plant Phys, 2020). Our conclusion is that mRNA processes are strongly affected within a few minutes after stress induction. For example, 25% of the transcriptome of Arabidopsis seedlings is targeted for rapid degradation only 15 minutes after 38°C heat stress (Merret et al., Cell Reports 2013). We chose to examine polysomal processes 15 min after stress because we estimate that this time point will capture a maximum of mRNA-related regulatory changes that occurred when switching from the normal (20°C) growth and developmental program to the 37°C and/or 44°C heat stress response program. We estimate that mRNA post-transcriptional regulation is mainly important for the rapid reprograming of gene expression, and by placing ourselves at the end of the two stress periods (1h for the 37°C stress and 30 min for the 44°C stress) one runs the risk of missing most of the very early events.

2. Have they ever considered why mRNA transcripts of different genes regulated differently during these polysome-related processes? I think an addition of related analysis or, at least in depth discussion in this point should improve the quality of the manuscript a lot. This is of course a very important (and huge) question. One of the major original contributions of our work is to provide, for the first time, strong evidence that CTRD is not a passive process whose level is always proportional to the amount of mRNA associated with polysomes but should be considered as an independent gene regulatory mechanism that can target some mRNAs more efficiently (Figure 5), resulting in differences in corresponding protein accumulation (Figure 6). In Figure 5, we have identified 1,126 gene transcripts that are only regulated by CTRD in at least one step of the SAT and/or BT heat stress regime. The heat map in Figure 5A shows that these transcripts can be separated into four clusters. For one of these clusters (cluster 4), the reason why these transcripts are major targets of CTRD at 44°C without priming (in BT) is presented in the paper and is probably due to the fact that they encode proteins with highly hydrophobic nascent peptides produced in the ER and that, without priming, the UPR response is not activated. Therefore, ER-specific HSP70s are not induced under these conditions, leading to ribosome arrest and induction of CTRD. This does not happen in SAT as the priming step allows the induction of UPR genes including ER-specific HSP70s (Figure 4 and Supplementary Table 2). For the transcripts in the other three clusters (1 to 3), the question remains open as they do not share the characteristics of the cluster 4 transcripts. One possibility is that the tRNA populations, as well as their epitranscriptomic status, vary during SAT and BT and that translation of transcripts with specific codon bias is slowed down (and CTRD increased) at some points in these heat stress regimes. We plan to do a large-scale analysis of tRNA steady-state levels and epitranscriptomic status (using mim-tRNA-seq pairing with mass spectrometry analysis) to test this hypothesis, but this is out of the scope of this paper. We have modified the discussion section of the paper to present this hypothesis.

Reviewer #3 (Comments for the Author):

In this study, Dannfald et al. conducted RNA-seq, polysome profiling, and 5'P seq analyses to elucidate plant responses to heat stress at the translational level with or without a priming event. The authors discovered that the priming event aids in restoring mRNA translation post-heat stress, and heat stress-responsive mRNAs exhibit increased translation efficiency compared to non-heat-responsive genes under heat-stressed conditions. Furthermore, the priming event triggers a set of genes related to the UPR pathway, while ER-translated mRNAs undergo cotranslational mRNA decay in the absence of priming. However, the manuscript is overly descriptive and lacks clarity, making the findings less appealing for publication in Plant Physiology.

Several concerns regarding the manuscript are as follows:
1) The calculation of translation efficiency is a major concern. The authors define TE as the ratio of polysomal mRNA-seq to degradome (5'P-seq) data, which may not accurately reflect TE. It is suggested that TE should be calculated as the difference between polysomal mRNA-seq and degradome over mRNA-seq, i.e., (polysomal mRNA-seq - degradome) / mRNA-seq. This discrepancy raises questions about the accuracy of TE calculation.

Calculating translation efficiency is not a trivial task. Since the discovery of the co-translational decay process, it is obvious that the simple detection of an mRNA molecule in polysome is not a clear indication of active translation, as it could be in the process of being degraded. It is not possible to simply subtract the reads obtained from the degradome database from the reads

obtained from the polysome database, since these reads are not absolute values, but are normalized values (TPM) representing a proportion of the reads. This proportion depends on the depth of the sequencing experiment and the complexity of the mRNA populations, factors that vary between the two types of databases. To compare polysome and degradome data, only TPM ratios are meaningful. We (in Carpentier et al., Plant Physiology, 2020:1251-1262) have clearly shown that using a ratio of the normalized amount of mRNAs associated with polysomes to the normalized amount of mRNAs involved in the co-translational decay process is a very good proxy for translation efficiency and corresponds very well to the final amount of protein produced. This is therefore the method we have used to calculate translation efficiency in this paper.

2) The relationship between RNA-seq, polysome profiling, and 5'P seq meta-analyses is not clearly explained in the manuscript, leading to confusion about how these analyses are interconnected.
We now provide two new Supplementary Figures (2 and 3) to clarify our filtering procedure and the relationship between our three NGS approaches.

3) Experimental validation of proposed TE or CTRD changes is missing from the manuscript, making the findings less robust.

We now provide new experimental evidence that changes in CTRD levels at 44°C between SAT (primed) and BT (unprimed) conditions can lead to corresponding changes in protein levels during the recovery period of these two heat stress regimes (Figure 6). Indeed, using specific antibodies, we observed that four cluster 4 mRNAs that are significantly more cotranslationally degraded in p4 (BT, without priming) compared to pC (SAT, with priming) produce less corresponding protein in BT (compared to SAT) during the recovery period. Since these cluster 4 mRNAs show no significant variation in global or polysomal steady-state levels, but only in the degradome database, we believe that these new experimental data provide new evidence for one of the main conclusions of this paper, that CTRD can regulate gene expression independently of other mechanisms.

4) According to the Figure5F and G, the abundance of XRN4 is increased under BT conditions, correspondingly, CTRD would be stronger. However, in Figure 3A, the situation contradicts with the hypothesis, need clarification to address inconsistencies in the data.

We understand that these two results may seem contradictory. Figure 3A is a meta-analysis of 13,123 mRNAs showing that they are globally slightly less co-translationally degraded at 44°C (primed and unprimed) compared to 20°C. This is in contrast to the fact that the major enzyme involved in co-translational decay (XRN4) is more abundant in polysomes at 44°C. However, we clearly show in Figure 5 that co-translational decay at 44°C is mainly targeted to a small subset of mRNAs (n=1,126), a very small fraction compared to the 13,123 mRNAs analyzed in Figure 3A. Therefore, XNR4 at 44°C is likely to act mainly on a small subset of mRNAs, which is why it is not reflected in the meta-analysis in Figure 3A. A clear example of this is the mRNAs belonging to cluster 4 (n=258, Figure 5A-B), which are much more degraded at 44°C without priming, as are all mRNAs associated with the GO term 'endoplasmic reticulum' (Figure 5C). Therefore, while the majority of mRNAs are not co-translationally degraded more at 44°C, a small proportion are, and XRN4 is likely to be specifically targeted to this subset, explaining its higher level detected in polysomes at 44°C.

5) Exploring mutants of HSP70 and XRN4 to analyze changes in TE efficiency and CTRD could enhance the manuscript's quality.

The ER HSP70s BIP1 and BIP2 have redundant functions and knockout of both genes is lethal (Maruyama et al., PNAS (2010) 107: 1684-1689), preventing the use of this strategy to study changes in TE and CTRD.

Although XRN4 is the major enzyme responsible for CTRD, we recently observed that other enzyme(s) are likely also involved (Carpentier et al., NAR (2024) https://doi.org/10.1093/nar/gkae363). We have also observed that in the *xrn4* mutant background, a feedback mechanism can occur on certain mRNAs to compensate for the loss of XRN4 and maintain (and sometimes even increase) CTRD (Carpentier et al., NAR (2024) https://doi.org/10.1093/nar/gkae363). Therefore, using the *xrn4* mutant to analyze changes in CTRD will not be trivial and is likely to vary according to mRNA populations. A better characterization of this feedback mechanism and the identification of this (or these) other enzyme(s) are research projects we are pursuing.

Minor concerns:
1) In the SAT conditions, the priming treatment lasts for 1 hour. Why did the authors collect tissues at 15 minutes after heat stress? Would it be more appropriate to collect tissues at the end of the priming event? A similar question arises for the P4 and PB conditions.

We have a lot of experience in studying mRNA processes after heat stress (Merret et al, Cell Reports 2013, Merret et al, NAR, 2015, Merret et al, Plant Phys, 2017, Carpentier et al, Plant Phys, 2020). Our conclusion is that mRNA processes are strongly affected within a few minutes after stress induction. For example, 25% of the transcriptome of Arabidopsis seedlings is targeted for rapid degradation only 15 minutes after 38°C heat stress (Merret et al., Cell Reports 2013). We chose to examine polysomal processes 15 min after stress because we estimate that this time point will capture a maximum of mRNA-related regulatory changes that occurred when switching from the normal (20°C) growth and developmental program to the 37°C and/or 44°C heat stress response program. We estimate that mRNA post-transcriptional regulation is mainly important for the rapid reprograming of gene expression, and by placing ourselves at the end of the two stress periods (1h for the 37°C stress and 30 min for the 44°C stress) one runs the risk of missing most of the very early events.

2) The figure legends should include more detailed information, particularly for the abbreviations used. For example, "GMUCT" in Figure 4 and the labeling in Figure 1B (4j? 8j?) should be clarified (e.g., 4d? 8d?). Additionally, statistical analysis should be provided for Figure 1B.

In this new version, we have ensured that the correct abbreviations are used and clearly defined in all figure legends. We now provide quantitative measures (and corresponding statistical analysis) of plant response after SAT and BT (Supplementary Figure1B).

3) There are numerous errors in the figures, such as Figure 3A where the values on the vertical axis are off-center. The descriptions like "* = p-value < 0.05, ** = p-value < 0.01, *** = p-value < 0.001" are not appropriate and should be revised.

This was corrected in the revised version

4) In Figure 4A, it displays 729 genes, while in line 310, it states "N=730." This inconsistency should be addressed and corrected.

729 is the correct amount, this inconstancy was corrected

5) There are discrepancies in the descriptions regarding data selection. For instance, in one section it mentions retaining mRNAs with at least 1 TPM in all conditions, while in another section, it refers to a filter applied to the original dataset of 37390 genes. These inconsistencies need to be clarified and resolved for coherence.

We now provide two new Supplementary Figures (2 and 3) to clarify our filtering procedure and the relationship between our three NGS approaches.

25-Oct-2024

Prof. Jean-Marc Deragon
1- CNRS LGDP-UMR5096, 58 Av. Paul Alduy 66860 PERPIGNAN, FRANCE. 2- Université de Perpignan Via Domitia, LGDP-UMR5096, 58 Av. Paul Alduy 66860 PERPIGNAN, FRANCE.
Biology
Perpignan 66860
France

RE: Plant response to intermittent heat stress involves modulation of mRNA translation efficiency

Dear Dr. Jean-Marc Deragon:

We are pleased to accept your manuscript for publication in the Plant Physiology. This acceptance is contingent on revision based on the comments of our reviewers. Please respond to Reviewer #4's comments (3rd review) by revising your manuscript to include additional relevant references, by clarifying, and expanding upon your results to address his/her concerns.

Please highlight all changes and include a detailed annotation to changes to the text, with line numbers, and noting your responses to the comments.

To submit your revised manuscript, click:

Link Not Available

If you cannot return the revised manuscript within 8 weeks of receipt, please let us know. Otherwise, we will assume that you have elected not to revise the manuscript and are withdrawing it.

Thank you for allowing us to review your work. We look forward to hearing from you soon.

Sincerely,

Editor, Plant Physiology

-- Reviewer comments:
Reviewer #1 (Comments for the Author):

Dear Authors,
Thank you for providing the suggested revisions based on my previous comments. The revisions to clarify the approaches and comparisons made are satisfactory. The supporting figures and revisions of figures that addressed some of the key issues raised are satisfactory. Most, if not all, of the grammatical errors were addressed. Overall, the revised manuscript incorporating all the reviewers' suggestions has significantly improved the interpretation of the results and conclusions.

Reviewer #2 (Comments for the Author):

The data provided support conclusion o this manuscript and the authors have answered comments well.

Reviewer #4 (Comments for the Author):

This manuscript by Dannfald et al. presents an elegant study showing how heat stress impacts polysome-bound RNA processes (namely, translation and co-translational decay). It is largely descriptive but presents some interesting observations and will be a useful community resource. I especially find interesting how rapidly (within 15 minutes) post-transcriptional (or polysomal) changes can be observed, which aligns with other recent papers (Smith et al 2024 The Plant Journal). The analysis method and alignment of molecular and biochemical observations to show how transcript-specific decay aligned with the levels of the encoding protein is also very nice (Figures 5 and 6).

However, I have some major concerns:
- My biggest concern is a potential fundamental flaw in the use of 5'P-seq as a proxy for co-translational RNA decay (CTRD).

Although a component, signal from 5'P-seq is the culmination of more than just CTRD. Instead, 5'P-seq has the most pronounced signal at miRNA cleavage sites (as was its original target e.g. German et al 2008 Nat. Biotech., Gregory et al 2008 Dev. Cell, Addo-Quaya et al 2008 Current Biology) as well as representing reads from decapped mRNAs (hence its modification to identify capping sites with "C-PARE" Nagarajan et al 2019 NAR), and mRNAs being decayed whilst bound by the exon junction complex before or during the pioneer round of translation (e.g. nonsense-mediated decay, see Lee et al 2020 and also summarized in Han et al 2023 The Plant Journal). There may also be lesser contributions of novel (or other hitherto unknown) endoribonucleases such as DNE1 (Nagarajan et al 2023 The Plant Cell, Pouclet et al 2024 The Plant Cell). I would propose two potential paths: (I) acknowledge this and reframe the manuscript to consider translation efficiency as polysome-associated RNA normalized by overall transcript-specific decay rate or (II) to alter the analysis such as to enrich the signal of CTRD in the 5'P-seq data. I assume the latter may be favored and the simplest way to do this may be to use the sum of counts (RPM) at bases 16 and 17 upstream of the stop codon (Hou et al 2016 The Plant Cell) instead of counts across the entire transcript. This approach may result in substantial filtering of transcripts as transcript-specific read coverage at these positions can be far lower than across an entire transcript. Therefore, the filtering of transcripts based on differential expression in mRNA-seq and poylsome mRNA-seq could also be re-examined and refined as necessary. Alternatively, use of metrics such as co-translation RNA decay index or terminal stalling index (Yu et al, 2016 The Plant Cell, Crisp et al 2017 The Plant Cell, Guo & Gregory 2023 Plant Direct) to define a set of CTRD mRNAs for which counts are considered across the transcript could be another alternative (and filtered for containing miRNA cleavage sites).

- The authors appear to argue that the standard in the field for translation efficiency is to consider polysomal RNA-sequencing alone. I must, respectfully, disagree. The majority of papers making claims around translation efficiency use the ratio of polysomal mRNA-sequencing / total mRNA-sequencing, as was defined by early work in Julia Bailey-Serres' group (e.g. Juntawong and Baily-Serres Frontiers in Plant Science 2012) and this has largely remained in more recent papers (e.g. Xu et al 2017 Nature, Chantarachot et al 2020 Nature Plants). I feel that the writing in the manuscript should reflect this. However, clearly, there appears to be good evidence to also consider decay or, perhaps, CTRD (this study, Carpentier et al Plant Physiology 2020). Perhaps, this would be an opportunity (e.g. in Figure 3) to contrast these different methods for determining TE (i) TE = polysome mRNA/ total mRNA, ii) TE = polysome mRNA/5'P-seq, iii) polysome mRNA) and see which better aligns with changes in protein levels. My impression is that the proposed method for TE here could supersede the current standard, however, I believe there needs to be an empirical comparison to do so and this was not done previously (Carpentier et al 2020 Plant Physiology).
- The missing experiment to convincingly show that CTRD is involved in thermal priming is to stress test the very interesting XRN4-CTRD line (Carpentier et al 2024 NAR) and observe whether it has impaired survivability under SAT in Figure 1 (more specifically, that its survivability is comparable between SAT and BT). For this, a phenotype would suffice rather than the exhaustive suite of molecular work. It would also be very interesting to see the overlap between XRN4-CTRD targets in Carpentier et al 2024 (NAR, shoot tissue, Figure 6A) with those defined as targets under heat stress (Figure 5, especially cluster 4).

Minor
- Please fix Supplementary Table 2, it is currently incompletely legible. Perhaps an excel spreadsheet is a more suitable format.
- It would be worthwhile to including the explanation provided to reviewers for the sampling timepoints when introducing the experimental schema. As the authors describe, this links back to their introduction that heat rapidly induces mRNA decay (alongside other work showing rapid mRNA changes in plants under stress e.g. Vogel et al 2014 The Plant Cell, Crisp et al 2017 The Plant Cell).
- I would be interested whether the authors considered applying the equation provided in Lee et al 2020 (The Plant Cell) for their metagene analysis presented in Figure 2. I would propose that using the normalized occurrence for 5'P-ends (and plotting their relative frequency) may highlight a greater difference between samples by accounting for transcript-specific differences in abundance (either as a result of varying transcription and/or decay). Simply averaging RPM across the transcriptome likely biases the signal to higher abundance transcripts.
- Could the authors please clarify how TPM (reads / length of transcript kb / per million scaling factor) was calculated per-nucleotide in Figure 2? Is it effectively RPM (reads / per million scaling factor)?

Answers to reviewer 4's comments:

- My biggest concern is a potential fundamental flaw in the use of 5'P-seq as a proxy for co-translational RNA decay (CTRD). Although a component, signal from 5'P-seq is the culmination of more than just CTRD. Instead, 5'P-seq has the most pronounced signal at miRNA cleavage sites (as was its original target e.g. German et al 2008 Nat. Biotech., Gregory et al 2008 Dev. Cell, Addo-Quaya et al 2008 Current Biology) as well as representing reads from decapped mRNAs (hence its modification to identify capping sites with "C-PARE" Nagarajan et al 2019 NAR), and mRNAs being decayed whilst bound by the exon junction complex before or during the pioneer round of translation (e.g. nonsense-mediated decay, see Lee et al 2020 and also summarized in Han et al 2023 The Plant Journal). There may also be lesser contributions of novel (or other hitherto unknown) endoribonucleases such as DNE1 (Nagarajan et al 2023 The Plant Cell, Pouclet et al 2024 The Plant Cell). I would propose two potential paths: (I) acknowledge this and reframe the manuscript to consider translation efficiency as polysome-associated RNA normalized by overall transcript-specific decay rate or (II) to alter the analysis such as to enrich the signal of CTRD in the 5'P-seq data. I assume the latter may be favored and the simplest way to do this may be to use the sum of counts (RPM) at bases 16 and 17 upstream of the stop codon (Hou et al 2016 The Plant Cell) instead of counts across the entire transcript. This approach may result in substantial filtering of transcripts as transcript-specific read coverage at these positions can be far lower than across an entire transcript. Therefore, the filtering of transcripts based on differential expression in mRNA-seq and poylsome mRNA-seq could also be re-examined and refined as necessary. Alternatively, use of metrics such as co-translation RNA decay index or terminal stalling index (Yu et al, 2016 The Plant Cell, Crisp et al 2017 The Plant Cell, Guo & Gregory 2023 Plant Direct) to define a set of CTRD mRNAs for which counts are considered across the transcript could be another alternative (and filtered for containing miRNA cleavage sites).

We agree with the reviewer that 5'P-seq analysis can capture degradation products that are not associated with polysomes, but we estimate that, particularly in a wild type (i.e. XRN4 functional) background, co-translational degradation products are largely dominant in this database, justifying its use to measure translation efficiency. To confirm this, we now provide ***a new supplementary figure (Supplementary Figure 5)*** *that present a comparison of the distribution of general 5'P-seq reads across the SAT and BT with the distribution of 5'P-seq reads found precisely at position 16-17nt before the stop codon (reads that, as the reviewer mentions, can only be rigorously attributed to CTRD). To do so, as suggested by the reviewer, we used the translational termination stalling index (TSI) defined as the ratio of the number of 5'P read ends at the ribosome boundary (16–17 nt upstream from stop codon) to the mean number of 5'P read ends within the flanking 100 nt (this index was used in several previous publications including Guo et al., 2023; Carpentier et al., 2024). We show that the distributions generated using the two datasets follow the same trend, confirming that CTRD products are dominant in the global 5'P-seq database in a wild-type (i.e. XRN4 functional) background. The text of the article has been modified to explain this situation, with the introduction of appropriate references* ***(lines 145 to 151, and 282 to 291)***

- The authors appear to argue that the standard in the field for translation efficiency is to consider polysomal RNA-sequencing alone. I must, respectfully, disagree. The majority of

papers making claims around translation efficiency use the ratio of polysomal mRNA-sequencing / total mRNA-sequencing, as was defined by early work in Julia Bailey-Serres' group (e.g. Juntawong and Baily-Serres Frontiers in Plant Science 2012) and this has largely remained in more recent papers (e.g. Xu et al 2017 Nature, Chantarachot et al 2020 Nature Plants). I feel that the writing in the manuscript should reflect this. However, clearly, there appears to be good evidence to also consider decay or, perhaps, CTRD (this study, Carpentier et al Plant Physiology 2020). Perhaps, this would be an opportunity (e.g. in Figure 3) to contrast these different methods for determining TE (i) TE = polysome mRNA/ total mRNA, ii) TE = polysome mRNA/5'P-seq, iii) polysome mRNA) and see which better aligns with changes in protein levels. My impression is that the proposed method for TE here could supersede the current standard, however, I believe there needs to be an empirical comparison to do so and this was not done previously (Carpentier et al 2020 Plant Physiology).

*We agree that the ratio of polysomal mRNAs to total mRNA is the usual standard used in the literature to measure translation efficiency (TE), and we have now changed our text to make this clear **(lines 154 and 258)**. However, measuring TE is not a trivial issue and we are not convinced that the perfect methodology has been found yet. Using the ratio of polysomal mRNAs to total mRNA is not really measuring TE per se, but rather the efficiency of translational commitment, which is not equivalent. For example, one can imagine a situation where there is a large pool of cytoplasmic mRNAs, but only a small fraction is associated with the ribosome. In this case, TE (measured as polysomal mRNAs to total mRNA) would be described as low. However, the small population of polysomal mRNAs could be actively translated and produce a significant amount of protein. Of course, the opposite situation could also occur (high engagement ratio but low ribosome processing and low protein production). Using polysome mRNA-seq divided by CTRD product, we can more directly measure the efficiency of the translation process once an mRNA is bound to a ribosome. This is because slowing down translating ribosomes is likely to induce CTRD. This is probably not the perfect method, but it has been shown to closely correspond to the level of protein production (in Carpentier et al. 2020 Plant Physiology). As suggested by the reviewer, below we compare in **A** the TE measurement as polysomal mRNAs to total mRNA to translation and in **B** TE measurement by polysomal mRNAs to CTR, through the SAT (P1 to P5) and BT (PB and PC) heat stress regimes.*

A

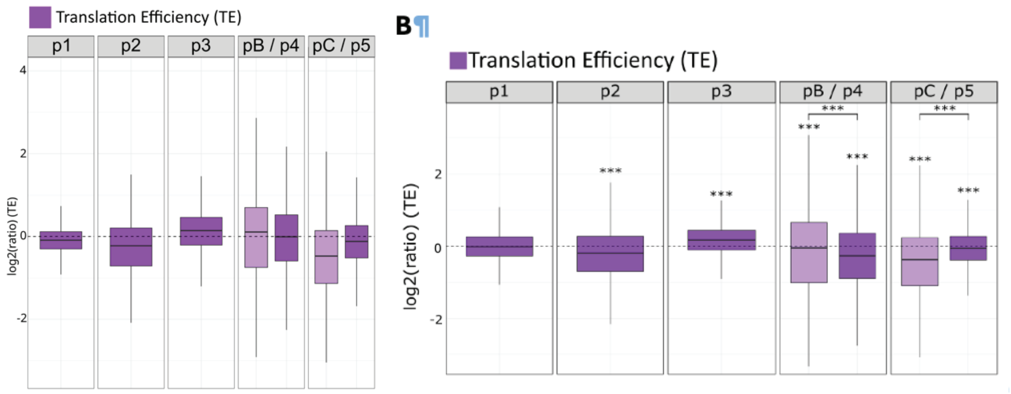

*These distributions are similar suggesting that translation engagement (**A**) and translation efficiency (**B**) are most of the time correlated.*

- The missing experiment to convincingly show that CTRD is involved in thermal priming is to stress test the very interesting XRN4-CTRD line (Carpentier et al 2024 NAR) and observe whether it has impaired survivability under SAT in Figure 1 (more specifically, that its survivability is comparable between SAT and BT). For this, a phenotype would suffice rather than the exhaustive suite of molecular work. It would also be very interesting to see the overlap between XRN4-CTRD targets in Carpentier et al 2024 (NAR, shoot tissue, Figure 6A) with those defined as targets under heat stress (Figure 5, especially cluster 4).

These are very interesting suggestions, but not as easy to implement as the reviewer suggests. We are currently working on these phenotypic aspects (extending our analysis to other heat stress regimes), but this work is beyond the scope of this paper. Regarding the overlap between the CTRD targets found in Carpentier et al. NAR 2024 and those found in this study, we must stress that the tissues used (shoot for the NAR study and whole seedlings in our case), the developmental stage (15 days old for NAR and 5 days old in our case) and the growth conditions (20°C for NAR and SAT/BT heat stress in our case) are very different. Nevertheless, we first made these comparisons using the 1126 CTRD targets we identified in our SAT/BT heat stress regimes (A) and, more specifically, the 258 genes in cluster 4 of Figure 5 (B)..

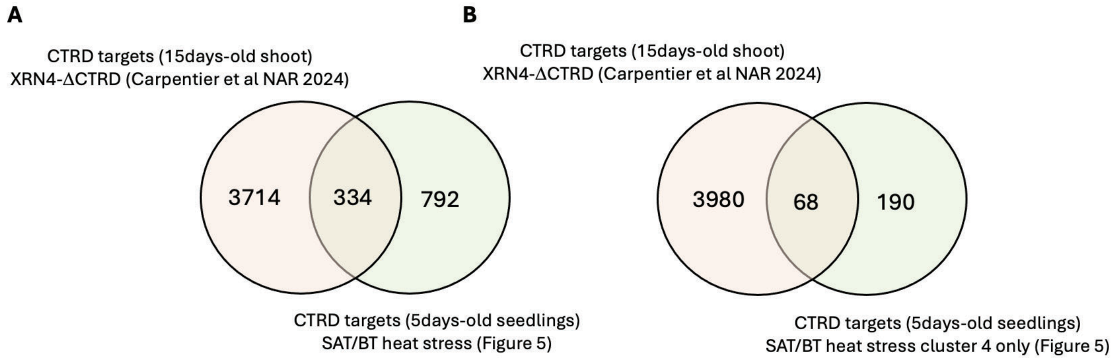

Despite significant differences between the two experimental settings, approximately 30% of the CTRD targets found in our heat stress setting (all or specific to cluster 4) were also identified as CTRD targets in the NAR study.

Minor

- Please fix Supplementary Table 2, it is currently incompletely legible. Perhaps an excel spreadsheet is a more suitable format.

We respectfully do not understand. Supplementary Table 2 is a normal excel spreadsheet that as been color-coded. maybe there was a problem with the extraction of this file?

- It would be worthwhile to including the explanation provided to reviewers for the sampling timepoints when introducing the experimental schema. As the authors describe, this links back to their introduction that heat rapidly induces mRNA decay (alongside other work showing rapid mRNA changes in plants under stress e.g. Vogel et al 2014 The Plant Cell, Crisp et al 2017 The Plant Cell).

*Thank you for this suggestion. We now have added this explanation **(lines 183 to 191)**.*

- I would be interested whether the authors considered applying the equation provided in Lee et al 2020 (The Plant Cell) for their metagene analysis presented in Figure 2. I would propose that using the normalized occurrence for 5'P-ends (and plotting their relative frequency) may highlight a greater difference between samples by accounting for transcript-specific differences in abundance (either as a result of varying transcription and/or decay). Simply averaging RPM across the transcriptome likely biases the signal to higher abundance transcripts.

We agree with the reviewer that the way we analyzed our 5'P-seq data in Figure 2 is somewhat biased towards higher abundance transcripts. However, the goal here is to document the effect of heat stress on global translation and compare it to the polysome profiles of Figure 1, which are also influenced by abundant transcripts. Therefore, the use of gene-per-gene normalization as proposed here is not appropriate for the comparison we want to make between Figure 1 and Figure 2, nor for the conclusion we want to draw (a global effect on translation).

- Could the authors please clarify how TPM (reads / length of transcript kb / per million scaling factor) was calculated per-nucleotide in Figure 2? Is it effectively RPM (reads / per million scaling factor)?

The reviewer is correct, we are indeed measuring RPM in Figure 2 and not TPM. This has been corrected. Sorry for this mistake.

11-Nov-2024
Prof. Jean-Marc Deragon
1- CNRS LGDP-UMR5096, 58 Av. Paul Alduy 66860 PERPIGNAN, FRANCE. 2- Université de Perpignan Via Domitia, LGDP-UMR5096, 58 Av. Paul Alduy 66860 PERPIGNAN, FRANCE.
Biology
Perpignan 66860
France

MSID: PP2024-RA-02392DR1
MS TITLE: Plant response to intermittent heat stress involves modulation of mRNA translation efficiency

Dear Dr. Jean-Marc Deragon:

I am pleased to inform you that your revised manuscript "Plant response to intermittent heat stress involves modulation of mRNA translation efficiency" has been accepted for publication in Plant Physiology. Your manuscript will be checked for consistency with journal formatting and image standards by one of our Science Editors, who will contact you shortly. They may have a number of queries and suggestions for you. Please keep in mind that they work to increase the online visibility of your article and its attraction for readers from the widest of backgrounds. You will have the opportunity to review any revisions, and I hope you will work constructively with them to get the most out of your research. You will also have the opportunity to review all changes when you receive the author proofs for your article.

Once validated for publication, your article will be sent to a Science Editor for a final review of your manuscript and source files. After your final files are uploaded, your article will appear in the next available issue and the online Preview version of your article will be posted after your signed Copyright and Checklist forms and final source files are received.

Please be sure to include the following in your manuscript file:
Harvard style citations
List of all author contributions and funding information at the beginning of manuscript after the titles and author list.
List all supplemental materials associated with the article, with titles, at the end of the ms, before the references and acknowledgments section.
Complete Figure Legends for all figures appearing in the article listed before the Literature Cited.

LICENSE INFORMATION Shortly after your paper is sent to production, you will receive an email from our publisher's author support team, **SciPris**, with information on article processing charges, as well as the types of Open Access license you would be able to purchase. The accepted version of your manuscript will not appear on our Advance Articles page until the license is selected and signed, so please look out for an email from SciPris. For more information see the Article Publication Fees section of our author guidelines: https://academic.oup.com/plcell/pages/General-Instructions

Finally, we encourage your submission of artwork for the journal cover. If there is an image or illustration related to your paper that you would like to have considered, please email it in .pdf or .tif format to clowe@aspb.org. An image title, brief, 2-3 sentence description of the image, and the name of the person credited for the image are also encouraged. If your image is selected to appear on the online cover of the journal, you will be contacted with the scheduled issue date and further information.

Thank you and congratulations on your Plant Physiology paper!

Sincerely,
Editor, Plant Physiology

=====
FIRST AUTHOR PROFILES
=====

We recognize the hard work that goes into being First Author of a Plant Physiology paper by publishing a "First Author Feature" for each First Author alongside your paper.

If the first author(s) would like to submit information for a feature on Facebook and Twitter, fill out the form by clicking on the link

below:

Plant Physiology First Author Feature Form: https://forms.office.com/r/ZBCGZfhm8F
If you cannot access the form through the link above, please contact Rachel at rbelsky@aspb.org.

To upload an image of yourself to accompany your profile, please put your photo in the correct journal folder at the link below. You must label your image: Author First Name Last Name Article Title.

First Author Profile Image Dropbox: https://www.dropbox.com/scl/fo/xz029p7qjr2jml2rp2kmq/h?dl=0&rlkey=6fkxcaqmxx35ap152b2osy1fz

=====
IMPORTANT REMINDER: PEER REVIEW REPORTS
=====

If you opted to publish a peer review report along with your article during the original submission process, it will be prepared by the editorial staff and publicly posted with your manuscript, inside the zip file that contains any other supplemental material. As a reminder, the peer review report is a public record of all comments from editors and reviewers, as well as your prior responses, as you received them in the decision letters for each draft of your manuscript. If you agreed to publish this report and have changed your mind, or are not sure if you selected this option, please contact the editorial office as soon as possible before signing the license agreement from our publisher.

-- Reviewer comments:
